# Supplementary material for: Interactive models of communication at the nanoscale using nanoparticles that talk to one another
Source: Nat Commun. 2017 May 30;8:15511. doi: 10.1038/ncomms15511 (PMC5459946; doi:10.1038/ncomms15511)
Supplement: Supplementary Information — Supplementary Figures, Supplementary Table, Supplementary Methods and Supplementary References. [file ncomms15511-s1.pdf]

Supplementary Methods

Chemicals. Tetraethyl orthosilicate (TEOS), *n*-cetyltrimethylammonium bromide (CTABr), sodium hydroxide (NaOH), tris(2,2′-bipyridyl)dichlororuthenium(II) hexahydrate ($[Ru(bpy)_3]Cl_2$), (3-iodopropyl)trimethoxysilane, benzimidazole, triethylamine, (3-mercaptopropyl)-trimethoxysilane, hydrogen tetrachloroaurate(III) ($HAuCl_4{\cdot}3H_2O$), sodium citrate tribasic dihydrate, paraffin wax, 3-mercatopropionic acid, potassium tert-butoxide, β-cyclodextrin, *N*-acetyl-L-cysteine, *N*-(3-dimethylaminopropyl)-*N*ˡ-ethylcarbodiimide hydrochloride (EDC), *N*-hydroxysuccinimide (NHS), β-galactosidase from *Aspergillus oryzae*, glucose oxidase from *Arpergillus niger*, lactose monohydrate, maltose monohydrate, lactulose, 2,2′-Azino-bis(3-ethylbenzothiazoline-6-sulfonic acid) diammonium salt (ABTS), peroxidase from horseadish (HRP) and D-glucose were purchased from Sigma-Aldrich. Sodium dihydrogen phosphate monohydrate, disodium hydrogen phosphate heptahydrate, sodium sulfate anhydrous and solvents were provided by Scharlau.

General methods. Powder X-ray diffraction (PXRD), transmission electron microscopy (TEM), N_2 adsorption-desorption isotherms, UV-visible spectrophotometry, thermogravimetric and elemental analysis, techniques were employed for materials characterization. PXRD measurements were performed on a Seifert 3000TT diffractometer using CuK_α radiation. TEM images were acquired using a JEOL TEM-1010 Electron microscope working at 100 kV. N_2 adsorption-desorption isotherms were recorded on a Micromeritics TriStar II Plus automated analyzer. UV-visible spectra were recorded with a JASCO V-650 Spectrophotometer. Thermogravimetric analysis were carried out on a TGA/SDTA 851e Mettler Toledo equipment, using an oxidant atmosphere (Air, 80 mL/min) with a heating program consisting on a heating ramp of 10 °C per minute from 393 K to 1273 K. Elemental analysis was performed in a CE Instrument EA-1110 CHN Elemental Analyzer.

Characterization of nanoparticles. Solids were characterized by standard techniques. Supplementary Figure 1 shows powder X-ray diffraction patters at low ($1.5 < 2\vartheta > 7$) and at high angles ($35 < 2\vartheta > 80$). At low angles, the as-made MCM-41 mesoporous silica nanoparticles (curve a) shows characteristic low-angle reflections. For the calcined **MCM-41** (curve b), we observed a slight displacement of the peaks related to the condensation of silanol groups during the calcination process. These low-angle typical peaks are preserved in the Au-MSN Janus colloids **S0** (curve c). The presence of the (100) peak in the PXRD patterns in

the solids **S1**, **S2**, **S1$_{gal}$** and **S2$_{gox}$** indicated that the different chemical modifications, functionalization and cargo loading had not damage the mesoporous structure. Moreover, the high-angle diffraction pattern of the all the Janus colloids showed the cubic gold characteristic (111), (200), (220) and (331) diffraction peaks, confirming the presence of gold nanocrystals and the Janus Au-MSN architecture.[1]

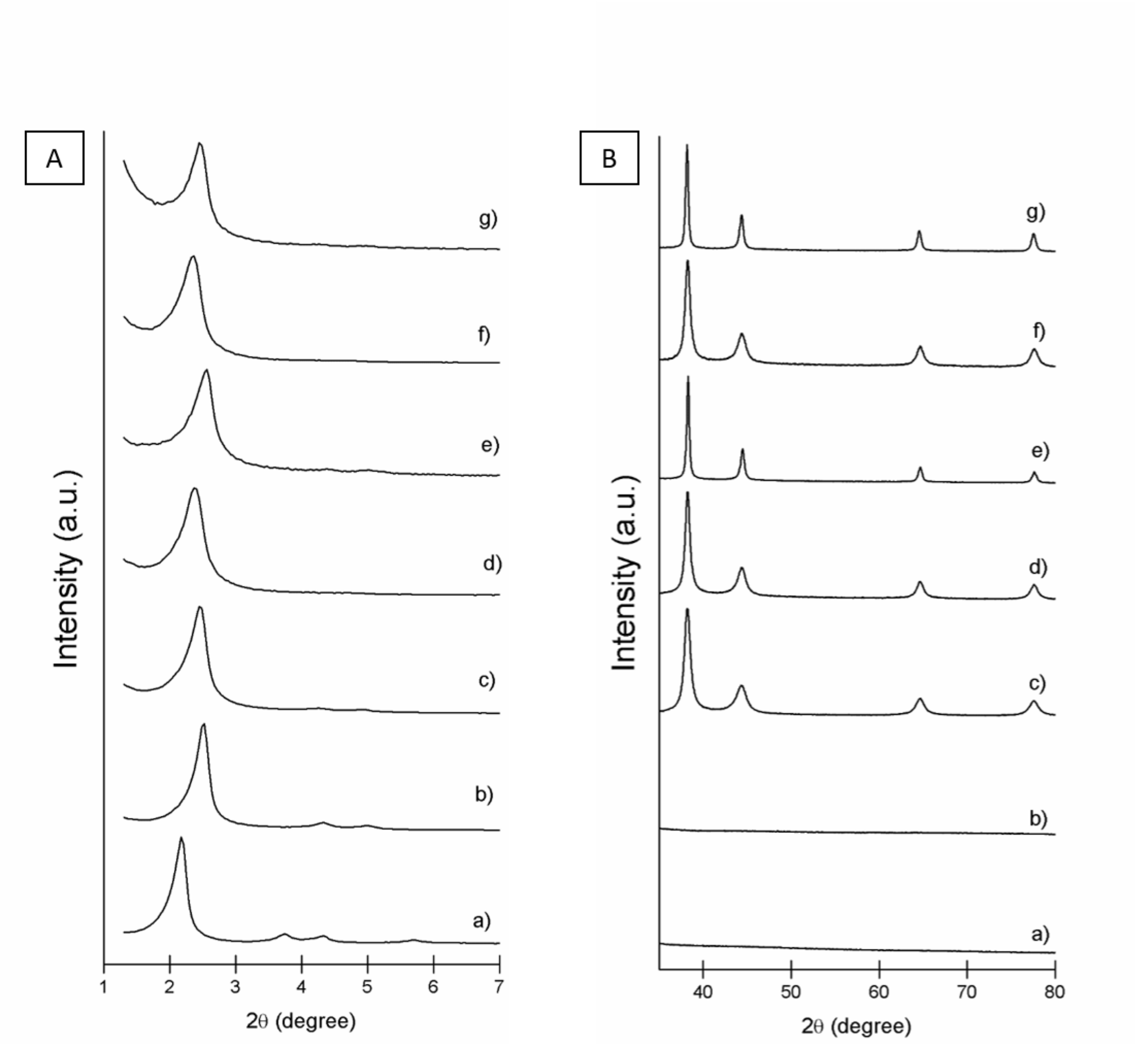

Supplementary Figure 1. Powder X-ray diffraction patterns of the solids (a) **as-made MCM-41**, (b) **calcined MSN**, (c) Janus Au-MS nanoparticles **S0**, (d) solid **S1**, (e) solid **S2**, (f) final nanodevice **S1$_{gal}$** and (g) final nanodevice **S2$_{gox}$** at low (A) and high (B) angles.

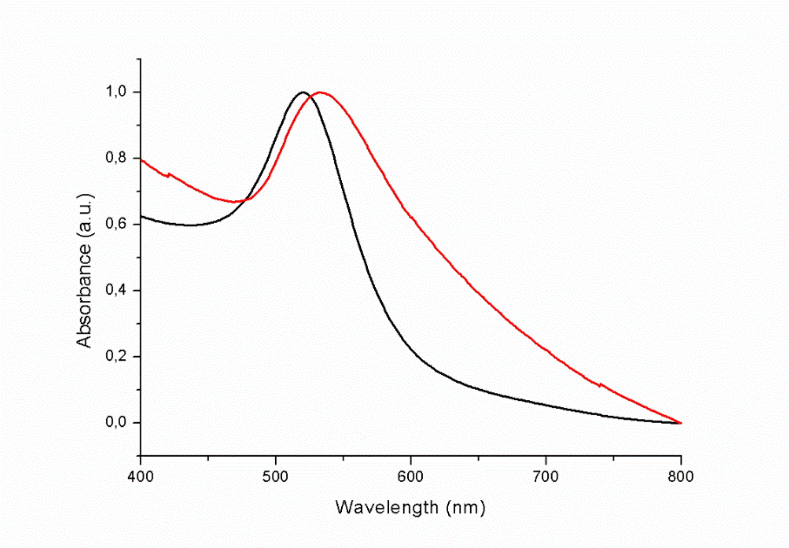

Supplementary Figure 2. Normalized UV-Visible spectra of the gold nanoparticles (black) and Janus Au-MS **S1** (red).

UV/vis measurements in aqueous solution were performed on the as-synthesized gold nanoparticles and on Janus Au-MS nanoparticles **S1** (by suspending 0.5 mg of solid in 1 mL of water). The starting gold colloid shows a single absorption band at 520 nm, characteristic of the surface plasmon resonance of spherically shaped nanospheres with an approximately 20 nm diameter (see Supplementary Figure 2). In the **S1** spectrum, there is a redshift of the absorbance maximum (535 nm) and a broadening of the band. These two facts can be ascribed to the increase in the refractive index around the gold nanospheres due to the MS attachment and to light refraction produced by silica.[2]

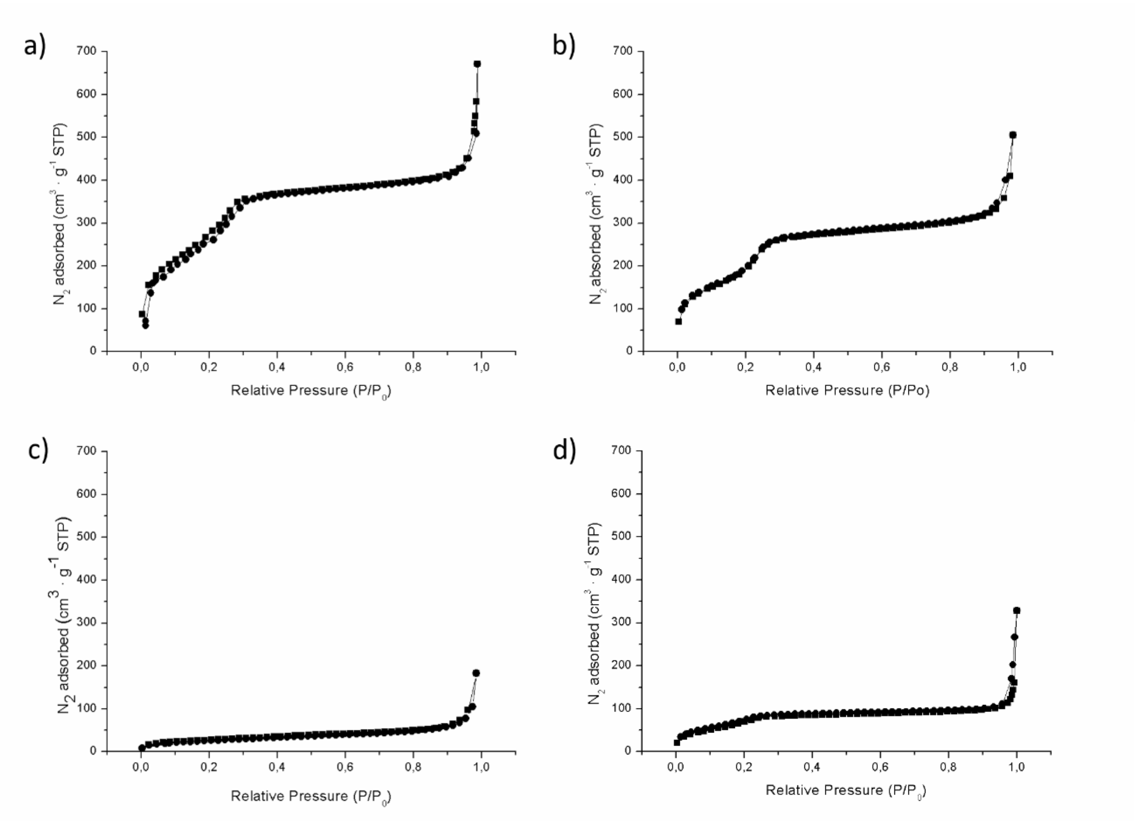

Supplementary Figure 3. The N_2 adsorption-desorption isotherms for (a) the calcined **MCM-41** mesoporous material, (b) Janus Au-MS nanoparticles **S0** and loaded and capped solids (c) **S1** and (d) **S2**.

The N_2 adsorption-desorption isotherms of the **calcined MCM-41** and Janus nanoparticles **S0** show an adsorption step at intermediate P/P_0 value 0.3, which is characteristic for mesoporous solids with empty pores (see Supplementary Figure 3). This step is related to the nitrogen condensation inside the mesopores by capillarity. The absence of a hysteresis loop in this interval and the narrow BJH pore distribution suggest the existence of uniform cylindrical mesopores. Application of the BET model results in a value for the total specific surface of 1093.9 $m^2 \cdot g^{-1}$ for calcined **MCM-41** and 879.1 $m^2 \cdot g^{-1}$ for **S0**. In contrast, N_2 adsorption-desorption isotherms for the functionalized and loaded solids **S1** and **S2** show a significant decrease in N_2 volume adsorbed and are flat when compared (at the same scale) to those from **MCM-41** and **S0**. This indicates that there is a significant pore blocking as a consequence of the loading and capping processes. BET specific values, pore volumes and pore sizes calculated from N_2 adsorption-desorption isotherms for **MCM-41**, **S0**, **S1** and **S2** are listed in Supplementary Table 1.

From the elemental analysis studies, the contents of $[Ru(bpy)_3]Cl_2$ and $-(CH_2)_3$-SS-CD on **S1** were determined as 119 mg and 78 mg per gram of solid, respectively. For **S2**, the *N*-acetyl-L-cysteine, benzimidazole and β-CD contents were determined as 31, 58 and 170 mg per gram of solid, respectively. This results correlate well with the thermogravimetric analysis in which a 20.4 % of organic content was determined for **S1** and 24.2 % for **S2**.

Supplementary Table 1. Elemental analysis for selected materials.

	%C	%H	%N	%S
**MCM-41**	--	0.60	--	--
**S0**	3.14	2.32	--	--
**S1**	13.39	2.49	1.56	4.04
**S2**	15.56	3.62	1.63	0.60

$Ru(bpy)_3Cl_2$ and $-(CH_2)_3$-SS-CD contents on **S1** were calculated from Supplementary Equations 1 and 2:

$$[Ru(bpy)_3Cl_2] = \frac{1.56\ g\ of\ N}{100\ g\ of\ S1} \times \frac{1\ mol\ of\ N}{14\ g\ of\ N} \times \frac{1\ mol\ of\ Ru(bpy)_3Cl_2}{6\ mol\ of\ N} \times \frac{640.53\ g\ of\ Ru(bpy)_3Cl_2}{1\ mol\ of\ Ru(bpy)_3Cl_2} = \frac{0.119\ g\ Ru(bpy)_3Cl_2}{g\ of\ S1}\ (\textbf{Supplementary Equation 1})$$

$$[-(CH_2)_3 - SS - CD] = \left(\frac{13.39\ g\ of\ C}{100\ g\ of\ S1} - \frac{3.14\ g\ of\ C}{100\ g\ of\ S0} - \frac{0.119\ g\ Ru(bpy)_3Cl_2}{g\ of\ S1} \times \frac{360\ g\ of\ C}{640.53\ g\ of\ Ru(bpy)_3Cl_2}\right) \times \frac{1\ mol\ of\ C}{12\ g\ of\ C} \times \frac{1\ mol\ of\ -(CH_2)_3{-}SS{-}CD}{45\ mol\ of\ C} \times \frac{1241\ g\ of\ -(CH_2)_3{-}SS{-}CD}{1\ mol\ of\ -(CH_2)_3{-}SS{-}CD} = \frac{0.082\ g\ of\ -(CH_2)_3{-}SS{-}CD}{g\ of\ S1}\ (\textbf{Supplementary Equation 2})$$

N-acetyl-L-cysteine (NAC), benzimidazole (BZI) and β-CD contents on **S2** were calculated from Supplementary Equations 3, 4 and 5:

$$[NAC] = \frac{0.60\ g\ of\ S}{100\ g\ of\ S2} \times \frac{1\ mol\ of\ S}{32\ g\ of\ S} \times \frac{1\ mol\ of\ NAC}{1\ mol\ of\ S} \times \frac{163.2\ g\ of\ NAC}{1\ mol\ of\ NAC} = \frac{0.031\ g\ NAC}{g\ of\ S2}\ (\textbf{Supplementary Equation 3})$$

$$[BZI] = \left(\frac{1.63\ g\ of\ N}{100\ g\ of\ S2} - \frac{0.031\ g\ of\ NAC}{g\ of\ S2} \times \frac{14\ g\ of\ N}{163.2\ g\ of\ NAC}\right) \times \frac{1\ mol\ of\ N}{14\ g\ of\ N} \times \frac{1\ mol\ of\ BZI}{2\ mol\ of\ N} \times \frac{118.14\ g\ of\ BZI}{1\ mol\ of\ BZI} =$$

$$\frac{0.058\ g\ of\ BZI}{g\ of\ S2}\ (\textbf{Supplementary Equation 4})$$

$$[\beta - CD] = \left(\frac{15.56\ g\ of\ C}{100\ g\ of\ S2} - \frac{0.031\ g\ NAC}{g\ of\ S2} \times \frac{60\ g\ of\ C}{163.2\ g\ of\ NAC} - \frac{0.058\ g\ BZI}{g\ of\ S2} \times \frac{72\ g\ of\ C}{118.14\ \ g\ of\ NAC} - \frac{3.14\ g\ of\ C}{100\ g\ of\ S0}\right) \times$$

$$\frac{1\ mol\ of\ C}{12\ g\ of\ C} \times \frac{1\ mol\ of\ \beta{-}CD}{42\ mol\ of\ C} \times \frac{1135\ g\ of\ \beta{-}CD}{1\ mol\ of\ \beta{-}CD} = \frac{0.170\ g\ of\ \beta{-}CD}{g\ of\ S2}\ (\textbf{Supplementary Equation 5})$$

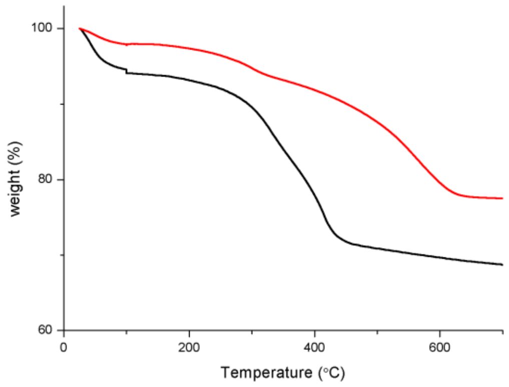

Supplementary Figure 4. Thermogravimetric analysis for **S1** (red curve) and **S2** (black curve).

Enzyme activity assays. The immobilization of the enzymes was confirmed by running the corresponding enzyme activity assays on each nanodevice. The method we used in order to test glucose oxidase activity is based on the oxidation of glucose by glucose oxidase which gives gluconic acid and hydrogen peroxide. Then, hydrogen peroxide reacts with ABTS in the presence of peroxidase to form a yellow product ($ABTS^{2-}$) that can be followed UV-visible spectrophotometry (Supplementary Equations 6 and 7).

Reactions for assaying glucose oxidase activity:

$$D - glucose\ +\ O_2 +\ H_2O \xrightarrow{glucose\ oxidase} D - gluconic\ acid + H_2O_2 (\textbf{Supplementary Equation 6})$$

$$H_2O_2 + ABTS \xrightarrow{peroxidase} ABTS^{2-} + H_2O\ (\textbf{Supplementary Equation 7})$$

In order to check glucose oxidase activity, 250 µL of 1 M of glucose (180 mg·mL^{-1}), 250 µL of ABTS solution (1 mg·mL^{-1}) and 50 µL of HRP solution (2 mg·mL^{-1}) were placed in a quartz cuvette. All solution had been prepared in 100 mM sodium phosphate buffer at pH 7.5. Then, 10 µL of either buffer (for blank) or $\textbf{S2}_{\textbf{gox}}$ suspension (7 mg·mL^{-1}) suspension were added. The

mixture was shaken and absorbance at 418 nm was monitored as a function of time. Whereas no change was observed in the absence of nanoparticles, a strong yellow colour appeared in the presence of those. The increase in absorbance ($ABTS^{2-}$ formation) as a function of time in the presence of **S2**$_{gox}$ is depicted in Supplementary Figure 5:

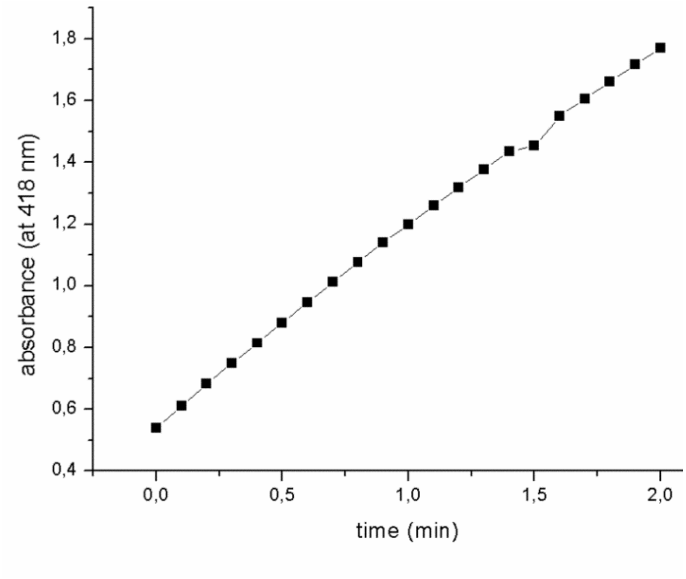

Supplementary Figure 5. Monitoring of $ABTS^{2-}$ formation due to glucose oxidase activity on **S2**$_{gox}$.

Glucose oxidase activity of **S2**$_{gox}$ was estimated to be 0.8 U·mg^{-1}, by applying the Supplementary Equation 8:

$$\frac{\text{Enzyme Units}}{\text{g}} = \frac{(\Delta - \Delta_{\text{blank}}) * V_T * F_D}{\varepsilon_{TNB} * \text{l} * V_{NPs} * C_{NPs}} \quad (\textbf{Supplementary Equation 8})$$

Where,

Δ is the slope of the graph (min^{-1})

Δ is the slope of the graph for the blank (min^{-1})

V_T is the total volume in the cuvette

ε_{TNB} is the molar extinction of $ABTS^{2-}$ at 418 nm (36.000 M^{-1} · cm^{-1})[3]

L is the optical path in the cuvette (1 cm)

V_{NPs} is the volume of nanoparticles added (mL)

C_{NPs} is the concentration of nanoparticles suspension added (g·mL^{-1}).

On the other hand, β-galactosidase activity on **S1**$_{gal}$ was checked by a similar protocol taking into account that lactose is hydrolyzed into glucose and galactose by β-galactosidase (see Supplementary Equations 9, 10 and 11).

$$lactose + H_2O \xrightarrow{\beta-galactosidase} D-glucose + galactose \text{ (\textbf{Supplementary Equation 9})}$$

$$D-glucose + O_2 + H_2O \xrightarrow{glucose\ oxidase} D-gluconic\ acid + H_2O_2 \text{(\textbf{Supplemantary Equation 10})}$$

$$H_2O_2 + ABTS \xrightarrow{peroxidase} ABTS^{2-} + H_2O \text{ (\textbf{Supplementary Equation 11})}$$

In order to check β-galactosidase activity, 250 µL of lactose solution (1 mg·mL^{-1}), 250 µL of ABTS solution (1 mg·mL^{-1}), 50 µL of glucose oxidase solution (1 mg·mL^{-1}) and 50 µL of HRP solution (2 mg·mL^{-1}) were placed in a quartz cuvette. Next, 10 µL of either buffer (100 mM sodium phosphate, pH 7.5) or **S1**$_{gal}$ suspension (8 mg·mL^{-1}) were added. The absorbance at 418 nm corresponding to the produced ABTS^{2-} was monitored. In this case, the β-galactosidase activity of **S1**$_{gal}$ was determined to be 0.5 U·g^{-1}. This smaller value when compared to the glucose oxidase activity on **S2**$_{gox}$ can be ascribed to the relatively low activity of commercial β-galactosidase. Using these procedures, the activity of commercial enzymes β-galactosidase and glucose oxidase were determined to be 30 U·g^{-1} and 115.7 U·mg^{-1} respectively.

Delivery studies from single nanodevices. Delivery experiments were carried out in order to study the envisioned capping-uncapping behavior with the aid of the dye-loaded nanoparticles **S1**$_{gal}$ and **S2**$_{dye}$ and UV-visible spectroscopy in the presence or in the absence of the corresponding molecular trigger.

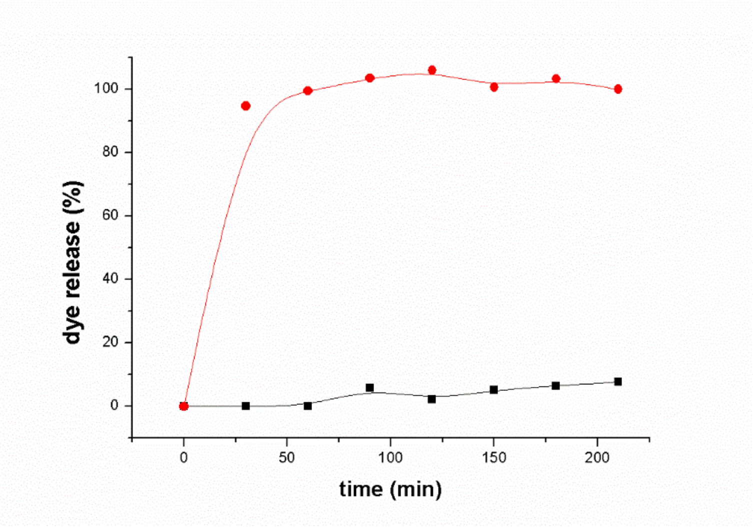

Supplementary Figure 6. Release of dye in aqueous solution at pH 7.5 from **S1**$_{gal}$ in the absence (black curve) and in the presence (red curve) of *N*-acetyl-L-cysteine (1 mM) monitored by measuring the $[Ru(bpy)_3]^{2+}$ absorbance at 453 nm.

In a typical experiment, a refrigerated solution **S1**$_{gal}$ was aliquoted, washed with freshly prepared aqueous solution at pH 7.5 (20 mM Na_2SO_4), divided in two fraction and finally brought to a concentration of 4 mg·mL^{-1} in aqueous media (20 mM Na_2SO_4, pH 7.5). Afterward, *N*-acetyl- L-cysteine (1 mM) was introduced to one of the suspensions and shaken over time. Aliquots were taken at scheduled times and centrifuged to remove the nanoparticles. Then, the absorbance at 453 nm corresponding to the $[Ru(bpy)_3]Cl_2$ released was measured. The results are shown in Supplementary Figure 6. From the kinetic curves it can be seen that solid **S1**$_{gal}$ was unable to release the cargo in absence on *N*-acetyl- L-cysteine, whereas in the presence of the input a remarkably cargo delivery was found.

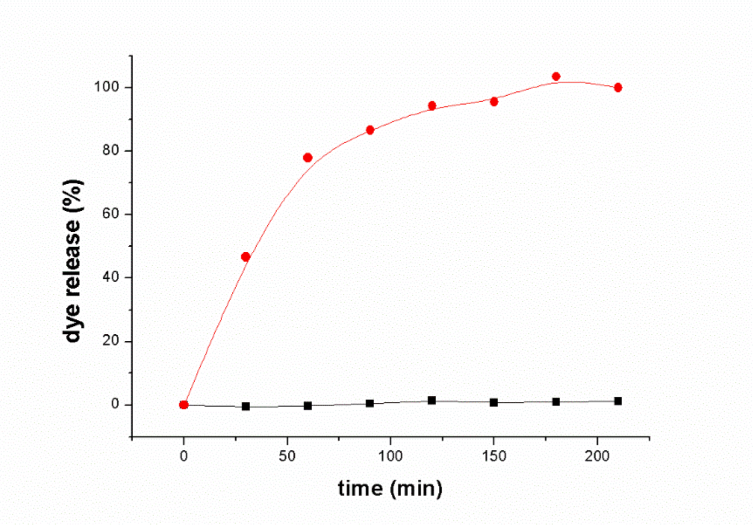

Supplementary Figure 7. Release of dye in aqueous solution at pH 7.5 from **S2**$_{dye}$ in the absence (black curve) and in the presence (red curve) of D-glucose (5 mM) monitored by measuring the ascribed $[Ru(bpy)_3]^{2+}$ absorbance at 453 nm.

Furthermore, delivery experiments were carried out using the capped material **S2**$_{dye}$ in the presence of D-glucose as trigger. In a typical experiment, a refrigerated solution of **S2**$_{dye}$ was aliquoted, washed with freshly prepared aqueous solution at pH 7.5 (20 mM Na_2SO_4), divided in two fraction and finally brought to a concentration of 1 mg·mL^{-1} in aqueous media (20 mM Na_2SO_4, pH 7.5). Then, D-glucose (5 mM) was introduced to one of the suspensions and shaken over time. Aliquots were taken at scheduled times, centrifuged to remove the nanoparticles, and the absorbance at 453 nm corresponding to the $[Ru(bpy)_3]Cl_2$ released was measured. As it can be seen in Supplementary Figure 7, in the absence of glucose a negligible dye release from **S2**$_{dye}$ was observed, whereas upon addition of D-glucose a remarkable release of cargo was found.

Supplementary References

1. Sánchez, A., Díez, P., Martínez-Ruíz, P., Villalonga, R. & Pingarrón, J. M. Janus Au-mesoporous silica nanoparticles as electrochemical biorecognition-signaling system. *Electrochem. Commun.* **30,** 51–54 (2013).
2. Kelly, K.L., Coronado, E., Zhao,L.L. & Schatz, G.C. The optical properties of metal nanoparticles: the influence of size, shape, and dielectric environment. *J. Phys. Chem. B* **107,** 668–677 (2003).
3. Shin, K-S. & Lee, Y-J. Purification and characterization of a new member of the laccase family from the white-rot basidiomycete Coriolus hirsutus. *Biochem. Biophys.* **384**, 109–115 (2000).
